# Bilateral Jaw Mycobacterium Abscessus Mimicking Actinomycosis: A Postoperative Complication of Wisdom Teeth Extraction

**DOI:** 10.7759/cureus.62336

**Published:** 2024-06-13

**Authors:** Michael Johanis, Karan S Cheema, Peter A Young, Gordon H Bae

**Affiliations:** 1 Department of Dermatology, Stanford University School of Medicine, Palo Alto, USA; 2 School of Public Health, Brown University, Providence, USA; 3 Department of Dermatology, Kaiser Permanente, Sacramento, USA

**Keywords:** wisdom teeth extraction, odontogenic infectionm skin infection, mycobacterium abscessus, bilateral jaw infection, actinomycosis israelii

## Abstract

The incidence of nontuberculous mycobacteria infections has surged over recent decades. *Mycobacterium abscessus* is one example that can present unique diagnostic challenges due to its variable antibiotic resistance profile and its clinical similarities to *Actinomycoses israelii* in postodontogenic infections. The authors report a case of a 22-year-old healthy female presenting with bilateral mandibular nodules following wisdom teeth extraction. After a presumptive diagnosis of actinomycosis, cultures revealed a *Mycobacterium abscessus* infection susceptible to macrolides. Magnetic resonance imaging depicted bilateral sinus tracts without osteomyelitis. The patient opted for dual antibiotic therapy, consisting of azithromycin and omadacycline, without surgical intervention. Given her clinical and radiographic improvement after three months, the patient elected to continue dual antibiotic therapy for 12 months with appropriate clinical and radiographic monitoring. This case underscores the importance of early microbial cultures to guide diagnosis and treatment, particularly considering *Mycobacterium abscessus*’s similarities with other pathogens and its variable macrolide susceptibility due to genetic mutations. As highlighted in this case, clinicians must successfully differentiate between and appropriately treat various nontuberculous mycobacteria.

## Introduction

There are over 150 species of nontuberculous mycobacteria (NTM), the incidence of which has increased significantly in the last few decades [1]. One study conducted at the Mayo Clinic found that the incidence of cutaneous NTM infection was about three-fold higher from 2000-2009 compared to that of 1980-1999 in the suburban county encompassing this institution; these increased rates can be attributed to a growing population of immunosuppressed patients, as well as an increase in cosmetic procedures, such as tattoos, body piercings, and liposuction [2,3]. In particular, *Mycobacterium abscessus* (*M. abscessus*), a gram-positive and acid-fast staining bacterium, is one rapidly growing NTM responsible for high rates of treatment failure due to its antimicrobial resistance [1]. *M. abscessus* can be misdiagnosed as *Actinomycoses israelii* (*A. israelii*) in postodontogenic infections, especially given the latter pathogen’s incidence following dental treatments. *A. israelii* is a gram-positive, non-acid-fast, and rod-shaped microbacterium that causes a chronic granulomatous infection [4,5].

## Case presentation

**Figure 1:**
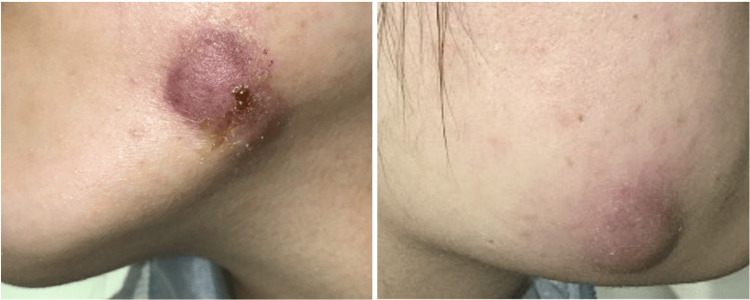
Initial clinical presentation of the superficial plaques located in the bilateral mandibular regions (A) An indurated superficial plaque at the patient’s left mandibular region that was incised and drained of pus, leading to crustation around the cyst. (B) A cyst on the patient’s right mandibular site, which was similarly incised and drained of pus.

A 22-year-old otherwise healthy female was referred for “bilateral inflamed cysts.” Initial evaluation showed erythematous to purpuric nodules located bilaterally about the mandible (Figures 1A, 1B). These lesions, which were firm, indurated, and painless, appeared four weeks after an uneventful extraction of her four wisdom teeth by an oral surgeon.

**Figure 2:**
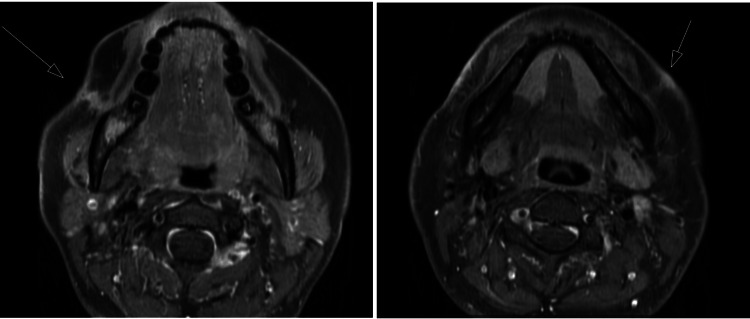
Initial MRI findings (A) A tract of edema and enhancement that extends to the skin surface, where there is mild puckering, thickening, and enhancement. (B) A linear tract of subcutaneous soft tissue edema and enhancement leading to the overlying skin surface, with mild skin thickening and enhancement but without puckering.

After a presumptive diagnosis of actinomycosis, an incision, and drainage were performed on both nodules to remove purulent tissue and material. Pathological examination revealed granulation tissue showcasing mixed inflammation, with no organisms seen on the gram stain. Tissue cultures were notable for *M. abscessus*, which was susceptible to macrolides without *rrl* or *erm* (41) mutations; Table 1 outlines the antibiotic susceptibility results. Magnetic resonance imaging (MRI) with contrast of the affected area revealed a sinus tract extending from the right buccal space at the level of the mandibular angle to the skin. A similar tract was noted on the left but to a lesser degree. Notably, there were no bony changes suggestive of osteomyelitis (Figures 2A, 2B).

**Table 1:** Mycobacterium Abscessus tissue culture’s susceptibility results

Antibiotic	Mycobacterium abscessus group (MIC MCG/ML)	
Amikacin	32 ug/mL	Intermediate
Cefoxitin	32 ug/mL	Intermediate
Ciprofloxacin	>4 ug/mL	Resistant
Clarithromycin	<=0.06 ug/mL	Susceptible
Doxycycline	>16 ug/mL	Resistant
Imipenem	16 ug/mL	Intermediate
Linezolid	2 ug/mL	Susceptible
Minocycline	>8 ug/mL	Resistant
Moxifloxacin	>8 ug/mL	Resistant
Tigecycline	0.25 ug/mL	No Interpretation
Trimethoprim/Sulfamethoxazole	>8 ug/mL	Resistant

**Figure 3:**
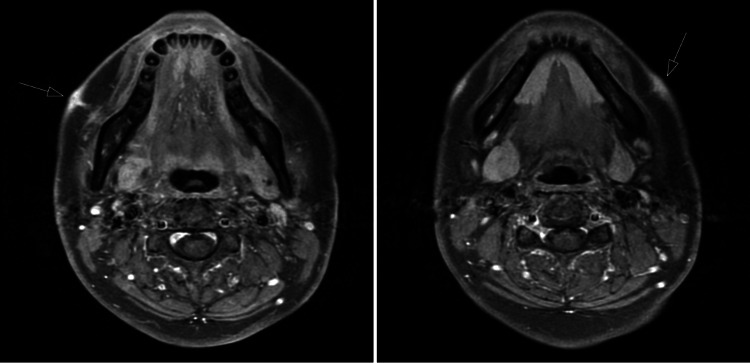
MRI findings at a three-month follow-up Notable improvement of the sinus tracts in the right buccal space and left perimandibular soft tissues following empiric antibiotic therapy, which, in combination with the patient’s preference to avoid surgery, significantly influenced the decision to continue antibiotic therapy for 12 months with appropriate clinical and radiographic monitoring.

The patient was prescribed azithromycin 250 mg daily and omadacycline 300 mg daily, and she was also referred to oral and maxillofacial surgery for possible sinus tract excision. She opted for antibiotic therapy without surgical intervention; given the risk of the infection spreading or failing to improve by declining surgery, the patient opted for serial monitoring every three months. Repeat MRIs in three months showed improvement of the sinus tracts in the right buccal space and left perimandibular soft tissues (Figure 3). Given the radiographic improvement, the patient opted to continue dual antibiotic therapy for 12 months with clinical and radiographic monitoring.

## Discussion

The differential diagnoses of postoperative odontogenic infections should include both *M. abscessus* and *A. israelii*. Endemic to the Southeastern USA (from Florida to Texas), *M. abscessus* is a rapidly growing mycobacterium that can be misdiagnosed as *A. israelii* [6]. This bacterium has been largely associated with dental procedures due to its prevalence in contaminated water, such as during irrigation procedures, though it is unclear whether our patient contracted the infection via a water source. One Southern California pediatric dental clinic reported in 2021 an outbreak of 71 cases, the largest outbreak of invasive *M. abscessus* at any pediatric health center [7]. There have been other reported outbreaks caused by non-sterile techniques and contaminated materials, particularly after surgical procedures including Mohs surgery, liposuction, breast tissue augmentation, and other cosmetic procedures [3]. Similarly, *A. israelii* is often associated with contaminated materials, but this pathogen can also cause cervicofacial actinomycosis, also known as “lumpy jaw syndrome,” which is present in 50-70% of infections [8]. Actinomycosis is rare, but the main risk factors are exposure to contaminated water during dental surgery or trauma, contact with contaminated sand, poor oral hygiene, malnutrition, and residence in tropical countries [8].

Since an accurate diagnosis of these infections can be difficult based exclusively on physical examination, we suggest obtaining microbial cultures early; with cultures, antibiotic susceptibility will also be available. Most *M. abscessus* subspecies *bolletii* and *M. abscessus* subspecies *abscessus* isolates have an active inducible macrolide resistance (*erm*) gene, which confers resistance to clarithromycin, though the *M. abscessus* subspecies *massiliense* does not. This difference affects treatment decisions regarding macrolide-involved regimens [9]. Additionally, acquired resistance to clarithromycin can be caused by a single point mutation in the *rrl* gene encoding its drug target, 23 rRNA, independent of an *erm* mutation. Numerous point mutations (T1406A, A1408G, and C1409T) have been identified in the rrs gene, which encodes 16S rRNA, conferring resistance to aminoglycosides. [10].

## Conclusions

The authors present a rare case of a bilateral jaw *M. abscessus* infection following wisdom teeth extraction. This case highlights the diagnostic utility of cultures, which in turn inform the treatment plan based on susceptibility results. Clinicians must be cognizant of the overlapping clinical features while being able to differentiate between and appropriately treat the two diseases.
